# Case report: Resolution of lameness via compartmental resection of a malignant nerve sheath neoplasm of the median nerve in a dog

**DOI:** 10.3389/fvets.2025.1551567

**Published:** 2025-02-13

**Authors:** Jeffery Smith, Marc Kent, Eric Glass, Garrett Davis

**Affiliations:** ^1^Red Bank Veterinary Hospital, Red Bank, NJ, United States; ^2^Department of Small Animal Medicine and Surgery, College of Veterinary Medicine, University of Georgia, Athens, GA, United States

**Keywords:** neuropathic pain, median nerve, malignant nerve sheath neoplasm, lameness, dog

## Abstract

A 7-year-old golden retriever was evaluated for a 6-month history of progressive right thoracic limb lameness. A lameness (grade 3 out of 5 on visual gait analysis) and pain with palpation of the medial aspect of the brachium proximal to the elbow were identified on exam. Magnetic resonance imaging of the right thoracic limb revealed a well-delineated, ovoid mass arising from the median nerve just proximal to the elbow. Compartmental resection of the mass with limb preservation was performed. Microscopically, the mass was a malignant nerve sheath neoplasm. One week postoperatively, the lameness was mild (grade 1). Three months postoperatively, the lameness had resolved (grade 0). One year postoperatively, the dog’s gait remains normal. Malignant nerve sheath neoplasms commonly arise in the brachial plexus or cervical spinal nerves, often affecting the innervation provided by the radial nerve. Given its role in providing weight support, dysfunction of the radial nerve significantly impacts the gait. Conversely, dysfunction of the median nerve should not impair the gait. In the present case, compartmental resection of the neoplasm affecting the median nerve resolved the dog’s lameness. The return of normal limb function supports the contention that the lameness was consequent to general somatic afferent dysfunction, neuropathic pain, rather than general somatic efferent function (paresis).

## Introduction

Causes of lameness in dogs are broadly divided into orthopedic and neurological etiologies. In orthopedic disorders, lameness occurs secondary to mechanical disruption of the limb movement, pain, or a combination of both. In neurological disorders, lameness occurs secondary to paresis, pain, or a combination of both. Regardless of the cause, the resultant lameness may look similar on visual gait analysis. In most cases, determining the underlying etiology requires physical examination and diagnostic imaging; however, it can be challenging if an apparent site of pain, mechanical dysfunction, or paresis is not readily identified on physical examination.

The following report details the diagnostic investigation and treatment of a dog with a chronic thoracic limb lameness. Examination alone failed to identify an orthopedic or neurologic etiology of the lameness. Clinicopathologic investigations and diagnostic imaging identified a malignant nerve sheath neoplasm (MNSN) involving the median nerve. With compartmental resection of the neoplasm, long-term resolution of the lameness and control of the neoplasm were achieved. The authors suggest that the dog’s lameness reflected neuropathic pain related to involvement of the median nerve. Compartmental resection with limb preservation was possible given the functional neuroanatomy provided by the median nerve and associated innervation.

## Case report

A 7-year-old, 35-kg, female spayed golden retriever presented for evaluation of a 5-month history of progressive right thoracic limb lameness. At the onset, the primary veterinarian appreciated a mild lameness on visual gait analysis (grade 1 out of 5) (1). No other abnormalities were identified on physical examination. A complete blood count and a serum biochemistry panel were normal. Radiographs of the right elbow disclosed mild sclerosis of the trochlear notch. Treatment with omega fatty acid supplements (Vetoquinol Triglyceride Omega, Fort Worth, TX, USA) failed to improve the lameness. At re-evaluation 6 weeks later, the lameness had worsened. Physical examination remained normal except for the right thoracic limb lameness. Point-of-care serology (SNAP 4Dx Plus Test, IDEXX Laboratories, Inc., Westbrook, ME, USA) for *E. canis* was positive; serology for *D. immitis*, *B. burgdorferi*, and *A. phagocytophilum* was negative. Follow-up PCR testing for *E. canis* was negative. Treatment with meloxicam (0.1 mg/kg orally q 24 h for 7 days) and doxycycline (10 mg/kg orally q 24 for 14 days) failed to improve the lameness. Two months after the onset of lameness, repeat radiographic evaluation of the right elbow was unchanged. Additionally, radiographs of the shoulder and carpus were obtained and were normal with the exception of mild antebrachiocarpal joint osteoarthritis. Four months after the onset of lameness, computed tomography (CT) of the right thoracic limb was performed at different referral hospital. Apart from sclerosis at the trochlear notch, no other abnormalities were reported. Injections of methylprednisolone acetate (20 mg per injection) into the tendon sheath of the biceps brachii tendon and into the area of the tendon of insertion of the supraspinatus muscle were performed. Despite methylprednisolone therapy, the lameness persisted. Five months after onset, the dog’s lameness continued to worsen and she was referred to Red Bank Veterinary Hospital for further evaluation of the lameness.

On presentation, a grade 3 out of 5 lameness of the right thoracic limb was observed. Pain was elicited with palpation of the medial aspect of the brachium proximal to the elbow. There also was moderate atrophy of the right thoracic limb musculature. With the exception of muscular atrophy, the remainder of the orthopedic examination was normal. Likewise, neurological examination was normal.

At the time of evaluation, only the CT imaging report was available for review. Since prior diagnostic imaging failed to reveal the cause of the lameness, magnetic resonance imaging (MRI) of the cervical vertebral column, right shoulder, and elbow was performed. Prior to MRI, a complete blood count, serum biochemistry profile, and a 3-view thoracic radiographic study were obtained. The blood work and thoracic radiographs were normal. Using a 1.5 -Tesla MRI unit (GE Brivo MR355 Inspire, General Electric Medical Healthcare Milwaukee, WI) multiplanar images were obtained of the cervical vertebral column and right shoulder. For cervical vertebral column and shoulder, sequences acquired included T2-weighted (T2W) and T1-weighted (T1W) prior to and following intravenous administration of contrast medium (0.2 mL/kg of gadolinium based contrast medium [0.2 mL/kg IV, Dotarem, Guerbet Princeton, NJ]). In the craniocaudal plane, a short tau inversion recovery (STIR) sequence was obtained of the right brachium that included the elbow and shoulder joints.

**Figure 1:**
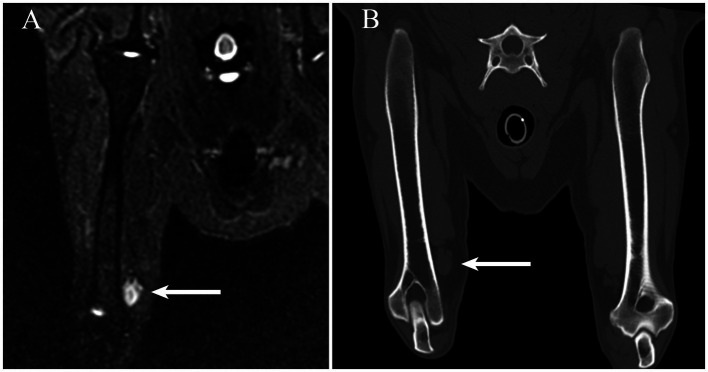
**(A)** On a short tau inversion recovery MRI sequence obtained in the craniocaudal plane, an ovoid hyperintense mass is readily identifiable adjacent to the medial aspect of the distal portion of the right humerus (arrow). **(B)** The mass (arrow) observed in panel **A** is visible on a multiplanar reconstruction CT image created along a plane similar to the short tau inversion recovery sequence.

In the right thoracic limb, a 1.5 cm × 5.0 cm ovoid subcutaneous mass was identified on the medial aspect of the distal brachium immediately adjacent to the brachial artery and vein. The mass was homogenously hyperintense on STIR images. (Figure 1) Subjectively, the right thoracic limb musculature was atrophied. The cervical vertebral column and right shoulder were normal. The imaging features were felt consistent with a neoplasm; however, a granuloma could not be excluded from consideration. Given the anatomic location, involvement of the median nerve was suspected. Muscular atrophy was considered secondary to disuse.

**Figure 2:**
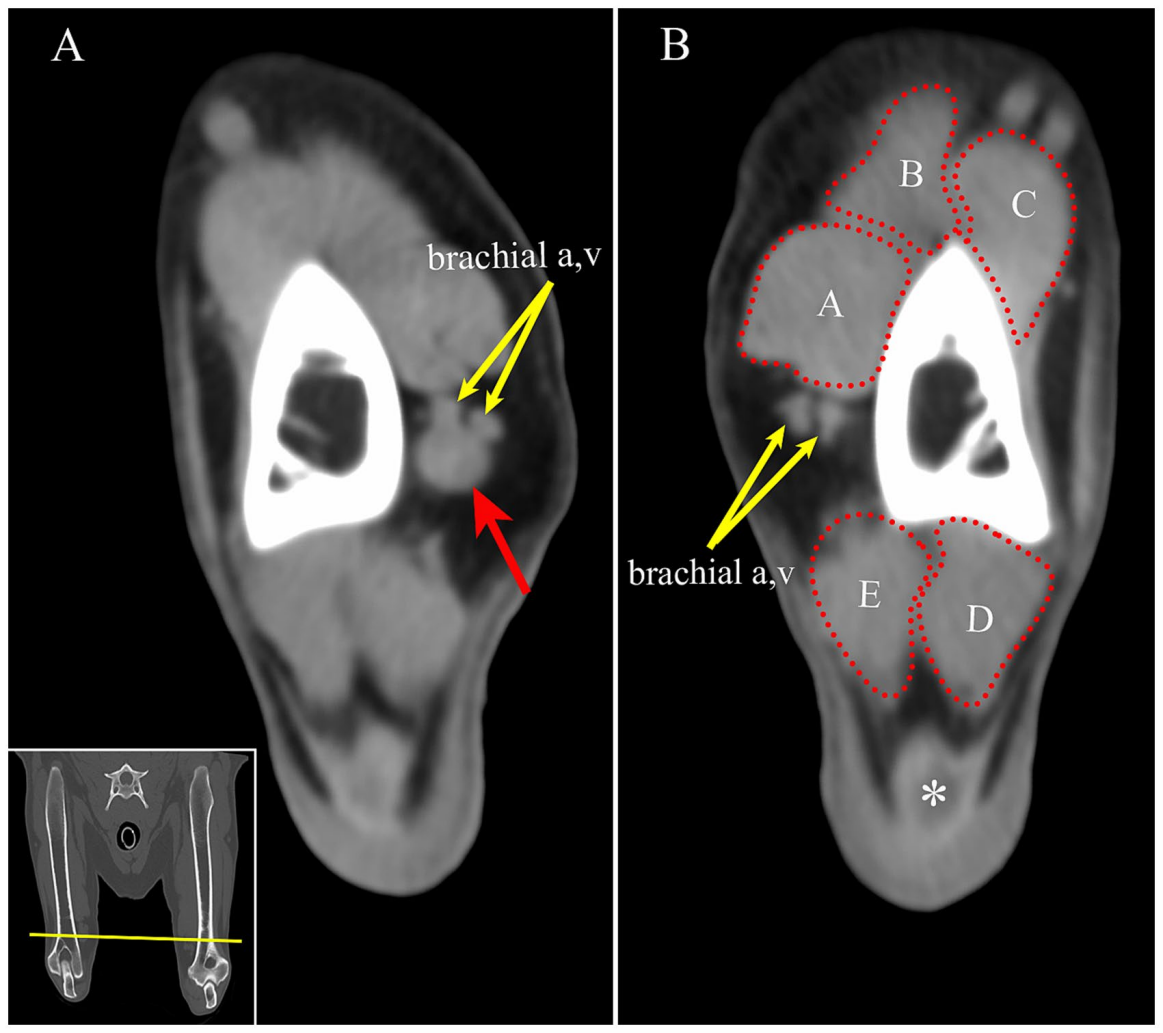
**(A)** On CT, the mass is identified as a round, soft tissue attenuating mass (red arrow) adjacent to the brachial artery and vein on the medial aspect of the right distal humerus (yellow arrows). **(B)** For comparison, the left brachial artery and vein are seen at approximately the same location on the left limb (yellow arrows). Transverse multiplanar reconstruction CT; acquired using a bone algorithm, window and level have been adjusted to make the mass most conspicuous. Inset: The yellow line is approximately the location of the transverse images in panel **A** and **B**. A-biceps brachii m. B-cleidobrachialis m. C-brachialis m. D- anconeus m. E- triceps brachii m, medial head, *- tendon of insertion of the triceps brachii muscle.

Having identified a mass on MRI, the prior CT study, which had been performed at a different institution, was obtained for review. At the same location as observed on the MRI, there was a well-defined, a soft tissue attenuating, ovoid mass (Figure 2).

In consultation with the owner, surgical exploration with the intent of compartmental resection of the mass and limb preservation was pursued. The following day, the dog was anesthetized. The hair overlying the medial aspect of the distal brachium and elbow was clipped and the skin was aseptically prepared. A 10 cm incision was made over the medial aspect of the distal brachium just proximal to the elbow. Deep to the subcutaneous fat and deep fascia, a focal mass involving the median nerve was identified. The collateral ulnar artery was isolated and ligated at the point where it crossed the median nerve. The communicating branch of the musculocutaneous nerve was identified and transected. Compartmental resection of the mass was achieved in which the median nerve was transected approximately 5 cm proximal and distal to the mass. The fascia and skin were closed routinely.

The excised mass and median nerve were submitted for histopathology. Microscopically, the nerve was expanded and effaced by a neoplastic population of indistinct, spindle-shaped cells. In some areas, the neoplastic cells formed whorls and fascicles. The neoplastic cells had variable amounts of eosinophilic cytoplasm and elongated, oval nuclei. The nuclei contained stippled chromatin with a single nucleolus. Occasional nuclear atypia was observed. Areas of necrosis and invasion through the perineurium also were present. Throughout the mass, there were areas of positive immunoreactivity for myelin protein zero which confirms involvement of the nerve. The microscopic findings combined with the immunohistochemistry were consistent with a MNSN. At the proximal and distal cut ends of the nerve, the neoplastic cells blended with the normal nerve cells. Consequently, radiation therapy was offered, however, the owner declined treatment.

Following surgery, the patient had a grade 2 out of 5 lameness on the right thoracic limb. The patient was discharged with carprofen (2.1 mg/kg orally q 12 h) and gabapentin (8.5 mg/kg orally q 12 h) for 14 days. One week post-operatively the owner reported improvements to the lameness.

At recheck 3 months and 8 months following surgery the dog’s gait was normal. Neurologic exam also was unremarkable. Cutaneous sensory testing of the palmar aspect of the paw was not performed. Via telephone communication with the owner, the dog continues to walk normally at 12 months postoperatively.

## Discussion

Lameness as a consequent to neurological dysfunction is the result of paresis/paralysis whereas lameness secondary to orthopedic disorders result in dysfunction of skeletal structures, muscles, tendons, ligaments, or joints. Neurologic and orthopedic disorders frequently are accompanied by pain which may further contribute to gait abnormalities. However, pain in the absence of an orthopedic or a neurologic disorder also may result in lameness.

Numerous classification schemes exist to categorize pain including those based on duration (acute or chronic), location, underlying disease (cancer pain), and mechanism involved (2). More broadly, pain can be classified as nociceptive or neuropathic (3). Nociceptive pain results from a noxious stimulus acting on a normal somatosensory nervous system (3). The somatosensory system, also known as general somatic afferent (GSA) system, is concerned with the perception of a noxious stimuli (nociception) as well as the perception of touch and temperature (4). In nociceptive pain, there is activation of normal receptors (nociceptors) in response to noxious stimuli. In short, nociceptive pain occurs secondary to damage or injury to non-neural tissue which evokes normal physiological activation of the somatosensory nervous system.

In contrast, neuropathic pain occurs secondary to disease or injury directly impacting either the peripheral or central nervous system components of the somatosensory system (3). In dogs, intervertebral disk herniation is commonly associated with neuropathic pain (2, 5). Persistent neuropathic pain may affect up to 15% of dogs following surgical treatment of intervertebral disk herniation (6). Moreover, neuropathic pain in dogs with intervertebral disk herniation may manifest as a nerve root signature (NRS) in which the affected dog is non-weight bearing in a standing position with possible concurrent lameness (7). In dogs with NRS secondary to caudal cervical spinal nerve root involvement, it is challenging to discern whether concurrent lameness is the result of neuropathic pain, general somatic efferent (GSE) dysfunction leading to paresis, or both.

In the present case, knowledge of the functional anatomy of the median nerve is imperative in understanding the etiology of the dog’s lameness. The median nerve arises primarily from the C8 and T1 spinal nerves with occasional contributions from the C7 and T2 spinal nerves (8). The median nerve provides both GSE and GSA innervation (8). Median nerve GSE axons innervate the pronator quadratus, pronator teres, flexor carpi radialis, deep digital flexor, and superficial digital flexor muscles (8). GSA axons, via the palmar branches of the median nerve, innervate cutaneous areas of the palmar aspect of the forepaw distal to the carpus (8). In the present case, GSE dysfunction can be excluded from playing a role in the cause of the lameness. Firstly, experimental transection of the median nerve in normal dogs does not produce an observable gait disturbance or lameness (9). Moreover, if GSE dysfunction contributed to the cause of the dog’s lameness, a worsening lameness postoperatively would have been anticipated as the median nerve was transected during compartmental resection of the neoplasm. In fact, the opposite occurred; the lameness completely resolved following resection of the neoplasm and transection of the median nerve proximal to the neoplasm along with transection of the communicating branch of the musculocutaneous nerve. Therefore, it is most plausible that with transection of median nerve, loss of GSA function abolished neuropathic pain secondary to the neoplastic infiltration of the nerve.

Ultimately, neuropathic pain can be challenging to identify. While subjective and objective methods of establishing pain exist, differentiating neuropathic pain from nociceptive pain is difficult in noncommunicating species. In the present case, neuropathic pain, as a result of neoplastic infiltration of the peripheral somatosensory nervous system, alone remains the most plausible explanation for the lameness. The MNSN affecting the median nerve fulfills the definition of neuropathic pain in that the median nerve conveys GSA information.

While neuropathic pain is often treated with analgesic medications, compartmental resection of the MNSN not only resolved the neuropathic pain as evidenced by a return of a normal gait but also constituted a definitive treatment. Conventionally, treatment of MNSNs in dogs frequently consists of local surgical excision and transection of the involved innervation combined with limb amputation (10–16) Treatment also may involve radiation therapy alone or following surgery (10, 12, 14–17). Depending on the extent of proximal invasion by the neoplasm, laminectomy may be required to excise neoplastic invasion within the intervertebral foramen, vertebral canal, or into the spinal cord, in an attempt to obtain margins histologically clear of neoplastic cells. Despite excessive surgical excision including laminectomy, achieving clear histological margins is difficult. Survival times with surgical excision and amputation vary widely and most reports do not provide the specific anatomic location of neoplasm along the course of the affected nerve(s). In general, dogs treated with local excision and amputation with or without radiation therapy, the median survival times vary from 120 days to 1,303 days with most studies reporting approximately 1 to 2 years (10, 12, 14–16, 18). In dogs with neoplastic extension into the intervertebral foramen, vertebral canal, or the spinal cord, the median survival time may be as short as 5 months, suggesting histological margins are important (14). In other studies, the influence of obtaining histologically clear margins is uncertain as clear margins did not significantly affect survival times (10, 12, 18). It is possible that such survival data is confounded by adjuvant radiation therapy (12).

In the present case, knowing the median nerve GSE function does not contribute to maintaining a normal gait, compartmental resection of the neoplasm with limb preservation was able to be performed. Conceptually, compartmental resection entails excision of the anatomical “compartment” that contains the neoplasm (19). In the setting of soft tissue sarcoma, the muscle or muscle groups containing the neoplasm are excised in an attempt to remove the neoplasm within the robust anatomic borders provided by the normal tissue rather than making arbitrarily sized borders (19). Compartmental resection with limb preservation may offer survival times similar to, if not longer, than excision and amputation (10, 12). Median survival time for dogs undergoing compartmental resection and limb preservation may be as long as 1,303 days (10). In the context of a MNSN, the “compartment” is likely considered the nerve itself. Consequently, neoplastic infiltration outside the epineurium may be an important determinant in considering compartmental resection with limb preservation. Neoplastic infiltration may confer higher recurrence rates and shorter survival times than non-infiltrative neoplasms (median survival time of 487 days vs. 2,227 days, respectively) (10). To date, the histologic diagnosis of MNSN can be difficult given variation in histological features of MNSN, histological similarities shared with other mesenchymal neoplasms, and the lack of specific immunohistochemical markers unique to MNSNs (20). Moreover, histological features do not impact survival following surgical resection (12).

Importantly, compartmental resection with limb preservation offers the potential of return of normal limb function. With compartmental resection, restoration of normal limb function occurs in approximately 60% of dogs with the remainder of dogs having residual lameness. Given the importance of the radial nerve to the maintenance of the gait, compartmental resection of neoplasms involving the radial nerve should be carefully considered as postoperative gait deficits are likely (10).

To the authors’ knowledge, four cases of MNSN involving the median nerve have been well documented (10, 11, 21, 22). The exact anatomic location of the neoplasm along the course of the median nerve was reported in three dogs (10, 11, 22). In two dogs the neoplasm was located at the level of the elbow; however, excision with amputation was performed in both dogs making comparison to the present case difficult (11, 21). In one dog, the neoplasm was located adjacent to the carpus and compartmental resection with limb preservation was performed (22). One month postoperatively, lameness was considered minimal (22). Further description of the dog’s gait was not provided. Limb amputation was performed at 26 months postoperatively due to recurrence (22). In the fourth case, the anatomic site of the neoplasm affecting the median nerve was not reported and the dog underwent compartmental resection with limb preservation (10). Although lameness improved postoperatively, the dog maintained a persistent weight-bearing lameness, experienced recurrence, and survived for 306 days (10). In contradistinction to the present case, reports of dogs treated with compartmental resection for MNSN of the median, lameness persisted postoperatively (10, 22).

Results achieved in the present case combined with previous reports suggest that compartmental resection of MNSN involving the median nerve with limb preservation is an acceptable treatment strategy providing survival times comparable to resection and limb amputation. Microscopic assessment of the proximal extent of the neoplastic involvement of the nerve should be performed to assess completeness of resection. Although experimental transection of the median nerve in normal dogs does not produce lameness, residual lameness may remain in dogs treated with compartmental resection for MNSN involving the median nerve (10, 22). While GSE dysfunction may play a role in postoperative lameness, new or persistent neuropathic pain may also contribute to the clinical signs. Future studies should investigate additional therapeutic interventions for the treatment of neuropathic pain in dogs having undergone compartmental resection that demonstrate residual neuropathic pain or lameness.

The present case report has several limitations. Chiefly, it is challenging to infer broad conclusions from a single case. Comparison to previously reported cases is hindered by differences in preoperative patient assessment, surgical techniques, and means of follow-up. While follow-up in the present case was collected via direct examination and telephone communication, imaging was not performed to assess the potential for recurrence at the surgical site. While recurrence may be present, maintenance of a normal gait suggests otherwise. Likewise, thoracic radiographs were not performed to assess for metastasis. Despite such drawbacks, the successful resolution of lameness postoperatively supports the notion that neuropathic pain may solely underlie the lameness observed in dogs with MNSN. Ultimately, the present case adds to the growing body of literature supporting the role of compartmental resection with limb sparing in providing long term survival for the treatment of MNSN in dogs.

## Conclusion

Dogs with malignant nerve sheath neoplasms frequently are presented for lameness as a result of paresis alone or in conjuction with neuropathic pain. Knowledge of the functional neuroanatomy of both the GSE and GSA innervations helps guide clinicians with treatment options. Although conventional treatment for MNSN involves excision combined with limb amputation, in cases involving innervation that has a limited impact on the ability to bear weight and walk, such as those involving the median nerve, compartmental resection with limb preservation may be considered. Even in cases involving radial nerve innervation in which residual deficits are expected, compartmental resection may improve lameness through alleviation of neuropathic pain (10). Postoperative care directed at control of neuropathic pain may result in additional improvement in limb function.

## Data Availability

The original contributions presented in the study are included in the article/Supplementary material, further inquiries can be directed to the corresponding author.

## Supplementary material

SUPPLEMENTARY VIDEO 1 Visual gait analysis at the time of presentation, a grade 3 out of 5 lameness of the right thoracic limb is present.

SUPPLEMENTARY VIDEO 2 3-month after compartmental resection of a malignant nerve sheath neoplasm of the median nerve, the dog’s gait is normal.

SUPPLEMENTARY VIDEO 3 An incision has been made on medial aspect of the right thoracic limb just proximal to the elbow. The median nerve has been isolated and retracted out of the surgical field using DeBakey hemostats. The closed tips of a Brown-Adson forceps trace the nerve from proximal (left side of the video) distally into a mass involving the median nerve. The smaller nerve that enters the mass is the communicating branch of the musculocutaneous nerve. In the second segment of the video, curved mosquito hemostats are used to grasp the proximal transected end of the median nerve to aide in the dissection of the mass distally (the elbow is toward the top left corner of the video).

## References

[1] Duerr F. Canine Lameness. Newark, NJ: John Wiley & Sons, (2020);3–13.

[2] Moore SA. Managing neuropathic pain in dogs. Front Vet Sci. (2016) 3:12. doi: 10.3389/fvets.2016.00012, PMID: 26942185 PMC4762016

[3] Merskey H Bogduk N. Part III: pain terms: a current list with definitions and notes on usage In: Merskey H Bogduk N, editors. Classification of chronic pain. Seattle: IASP Press (1994)

[4] de Lahunta A Glass E Kent M. General sensory systems: general proprioception and general somatic afferent, (Eds): Lahunta Alexander de Glass Eric Kent Marc, de Lahunta's veterinary neuroanatomy and clinical neurology. Philadelphia, PA: Elsevier, (2020);246–266.

[5] Fenn J Olby NJ. Classification of intervertebral disc disease. Front Vet Sci. (2020) 7:579025. doi: 10.3389/fvets.2020.579025, PMID: 33134360 PMC7572860

[6] Zidan N Medland J Olby N. Long-term postoperative pain evaluation in dogs with thoracolumbar intervertebral disk herniation after hemilaminectomy. J Vet Intern Med. (2020) 34:1547–55. doi: 10.1111/jvim.15800, PMID: 32462728 PMC7379041

[7] Schachar J Bocage A Nelson NC Early PJ Mariani CL Olby NJ. Clinical and imaging findings in dogs with nerve root signature associated with cervical intervertebral disc herniation. J Vet Intern Med. (2024) 38:1111–9. doi: 10.1111/jvim.16982, PMID: 38216520 PMC10937489

[8] Hermanson JW DeLahunta A Evans HE. Miller and Evans' anatomy of the dog. St. Louis, MO: Elsevier, (2020);704–756.

[9] Worthman RP. Demonstration of specific nerve paralyses in the dog. J Am Vet Med Assoc. (1957) 131:174–8. PMID: 13448982

[10] van Stee L Boston S Teske E Meij B. Compartmental resection of peripheral nerve tumours with limb preservation in 16 dogs (1995-2011). Vet J. (2017) 226:40–5. doi: 10.1016/j.tvjl.2017.07.002, PMID: 28911840

[11] Targett MP Dyce J Houlton JEF. Tumours involving the nerve sheaths of the forelimb in dogs. J Small Anim Pract. (1993) 34:221–5. doi: 10.1111/j.1748-5827.1993.tb02669.x

[12] Stokes R Wustefeld-Janssens BG Hinson W Wiener DJ Hollenbeck D Bertran J. Surgical and oncologic outcomes in dogs with malignant peripheral nerve sheath tumours arising from the brachial or lumbosacral plexus. Vet Comp Oncol. (2023) 21:739–47. doi: 10.1111/vco.12938, PMID: 37727977

[13] Kerwin SC Taylor AR. Neurologic causes of thoracic limb lameness. Vet Clin North Am Small Anim Pract. (2021) 51:357–64. doi: 10.1016/j.cvsm.2020.12.003, PMID: 33558012

[14] Brehm DM Vite CH Steinberg HS Haviland J van Winkle T. A retrospective evaluation of 51 cases of peripheral nerve sheath tumors in the dog. J Am Anim Hosp Assoc. (1995) 31:349–59. doi: 10.5326/15473317-31-4-349, PMID: 7552669

[15] Lacassagne K Hearon K Berg J Séguin B Hoyt L Byer B. Canine spinal meningiomas and nerve sheath tumours in 34 dogs (2008-2016): distribution and long-term outcome based upon histopathology and treatment modality. Vet Comp Oncol. (2018) 16:344–51. doi: 10.1111/vco.12385, PMID: 29363264

[16] Cooper-Khan RS Frankovich AN Thompson CA Thomovsky SA Lewis MJ. Clinical findings and outcome in 30 dogs with presumptive or confirmed nerve sheath tumors. Vet Sci. (2024) 11:11. doi: 10.3390/vetsci11050192, PMID: 38787164 PMC11125868

[17] Dolera M Malfassi L Bianchi C Carrara N Finesso S Marcarini S. Frameless stereotactic volumetric modulated arc radiotherapy of brachial plexus tumours in dogs: 10 cases. Br J Radiol. (2017) 90:20160617. doi: 10.1259/bjr.20160617, PMID: 27885855 PMC5605032

[18] Potamopoulou M Petite A Findji L. Combined forequarter amputation and hemilaminectomy for treatment of canine peripheral nerve sheath tumors of the brachial plexus invading the spinal canal: surgical technique and outcome in nine dogs. Vet Surg. (2024) 53:1477–84. doi: 10.1111/vsu.14166, PMID: 39315668

[19] Bray JP. Soft tissue sarcoma in the dog - part 2: surgical margins, controversies and a comparative review. J Small Anim Pract. (2017) 58:63–72. doi: 10.1111/jsap.12629, PMID: 28160303

[20] Tekavec K Švara T Knific T Gombač M Cantile C. Histopathological and Immunohistochemical evaluation of canine nerve sheath tumors and proposal for an updated classification. Vet Sci. (2022) 9:9. doi: 10.3390/vetsci9050204, PMID: 35622732 PMC9144584

[21] Anderson O Langley-Hobbs SJ. A peripheral nerve sheath tumour in the median nerve of a dog. Vet Rec Case Rep. (2022) 10:e323. doi: 10.1002/vrc2.323, PMID: 39834876

[22] Miyaho N Mochizuki M Honnami M. Forelimb lameness caused by malignant peripheral nerve sheath tumors of the median nerve in a dog: a case report. J Vet Med Sci. (2024) 86:860–5. doi: 10.1292/jvms.24-0038, PMID: 38945917 PMC11300132

